# Commentary: Standardization of procedures for health care providers safety in the coronavirus disease 2019 (COVID-19) era, with an eye to the future

**DOI:** 10.1016/j.xjtc.2020.12.028

**Published:** 2020-12-26

**Authors:** Marco Scarci, Federico Raveglia

**Affiliations:** Department of Thoracic Surgery, ASST Monza e Brianza, Ospedale San Gerardo, Monza, Italy

**Figure:**
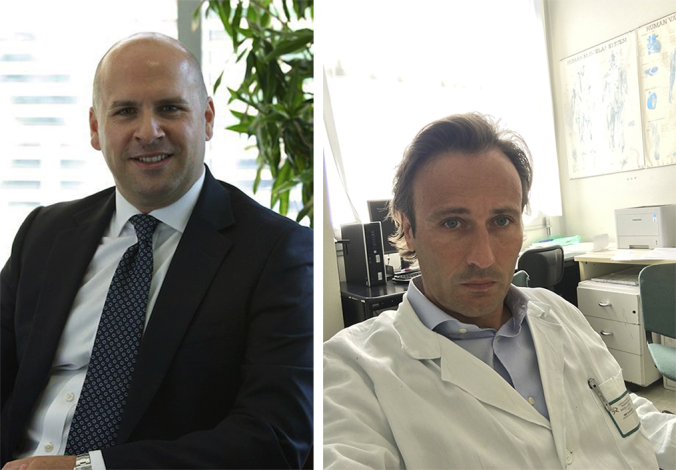
Marco Scarci, MD, FRCS(Eng), FCCP, FACS, FEBTS, and Federico Raveglia, MD

**Central Message**

Multidisciplinary approach and standardization of procedures deliver an improvement to patients and healthcare providers during COVID-19, but lessons can be learnt to enhance services after pandemia.

See Article page 183.

This article stimulated our interest for 2 reasons, as it reports both a pathway to safely perform tracheostomy and, more generally, suggests a model for surgical practice during and after the coronavirus disease 2019 (COVID-19) era.^1^ In the pandemic scenario, a tracheotomy is frequently indicated for patients affected by COVID-19 and severe acute respiratory syndrome. To our knowledge, guidelines showing the appropriate timing to perform a tracheostomy are not available and, also, waiting for a negative throat swab is not always feasible, especially during an emergency. Since tracheotomy exposes the airway, surgeons are directly exposed to the droplets and aerosol particles generated by the cough reflex, a procedure that carries one of the greatest risks of virus transmission.

Thal and coworkers^2^ have already reported a case series of 65 procedures in which they showed that infection risk for surgical providers is minimal if standard protective equipment is adopted (surgical gown, gloves, glasses or shield, hair cap, and N95 mask). Lee and coworkers^3^ focused on a protocol to protect surgeons and guarantee patient safety, considering that communication between staff members wearing personal protective equipment is challenging and, if an emergency happens, this cannot be appropriately managed unless all necessary devices are prepared in advance.

In this article, instead, the authors have remarkably emphasized that infective risk is a matter that concerns all operators who take part in the procedure and have adopted a multidisciplinary approach to determine a panel of recommendations aimed to protect all health care professionals. Interestingly, their method is based on a laboratory simulation and on a checklist-based approach.

It is our opinion that in modern medicine, patient management requires a multidisciplinary approach and standardization of procedures to guarantee the safety of operators and good outcomes for patients while saving resources at the same time. The advent of the pandemic has made these topics even more pressing. Therefore, starting from this manuscript, we would like to remark on 2 main issues. First of all, we suggest that the authors' method could be adopted to create guidelines for other invasive procedures in COVID-19 patient management. In particular, we refer to chest tube insertion, a procedure that is performed at the bedside on the ward or in the intensive care unit for pleural effusions or, more often, positive pressure–induced pneumothorax. In some studies, authors have already faced the issue of chest drain aerosol generation and COVID-19 infection.^4^ Second, we prompt readers to consider the unique opportunity offered by the pandemic to rethink clinical pathways to deliver service improvements and greater efficiency during the difficult recovery phase. To conclude, this paper is an excellent example of workflow reorganization during an emergency and for the upcoming periods after the pandemia.
